# Development of the Zambian Standard Treatment Guidelines in the Animal Health Sector: A Key Step in Advancing Antimicrobial Stewardship

**DOI:** 10.3390/antibiotics14111093

**Published:** 2025-11-01

**Authors:** Chikwanda Chileshe, Fusya Goma, Ntombi B. Mudenda, Steward Mudenda, Taona Sinyawa, Mwendalubi Hadunka, Geoffrey Mainda, Namukolo Muyamwa, Chrisborn Mubamba, Niwael Jesse Mtui Malamsha, Suze Percy Filippini, Maisa Kasanga, Victor Daka, Webrod Mufwambi, Amon Siame, Aubrey C. Kalungia, Zoran Muhimba, Mercy Mukuma, Sandra Diana Mwadesta, Shikanga O-Tipo, Jimmy Hangoma, John Bwalya Muma, Joseph Yamweka Chizimu, Charles Maseka, Roma Chilengi

**Affiliations:** 1 Zambia National Public Health Institute, Antimicrobial Resistance Coordinating Committee, Lusaka 10101, Zambia; steward.mudenda@unza.zm (S.M.); chizimuyjoseph@yahoo.com (J.Y.C.); chilengir@yahoo.com (R.C.); 2 Department of Clinical Studies, School of Veterinary Medicine, University of Zambia, Lusaka 10101, Zambia; ntombi.nkonde@unza.zm; 3 Ministry of Fisheries and Livestock, Lusaka 10101, Zambia; chinyemba08@gmail.com; 4 Department of Pharmacy, School of Health Sciences, University of Zambia, Lusaka 10101, Zambia; webrod.mufwambi@unza.zm (W.M.); chichokalungia@gmail.com (A.C.K.); 5 Department of Disease Control, School of Veterinary Medicine, University of Zambia, Lusaka 10101, Zambia; taonasinyawa@gmail.com (T.S.); jmuma@unza.zm (J.B.M.); 6 Center for Infectious Diseases Research in Zambia, Lusaka 10101, Zambia; mwendalubi.hadunka@cidrz.org (M.H.); amon.siame@cidrz.org (A.S.); 7 Food and Agriculture Organization of the UN, Lusaka 10101, Zambia; geoffrey.mainda@fao.org (G.M.); namukolo.muyamwa@fao.org (N.M.);; 8 School of Medicine, Copperbelt University, Ndola 10101, Zambia; 9 University Teaching Hospital, Lusaka 10101, Zambia; zmuhimba@yahoo.com; 10 School of Agriculture, University of Zambia, Lusaka 10101, Zambia; mercy.mukuma@unza.zm; 11 Word Health Organization, Lusaka 10101, Zambia; mwadesta@who.int (S.D.M.); otipos@who.int (S.O.-T.); 12 Pharmacy Department, Levy Mwanawasa Medical University, Lusaka 10101, Zambia; jimmyhangoma0282@gmail.com

**Keywords:** standard treatment guidelines, animal health, antimicrobial resistance, antimicrobial stewardship, Zambia

## Abstract

**Background**: Zambia, like many low- and middle-income countries, faces a growing burden of antimicrobial resistance (AMR), driven by the misuse of antimicrobials in both human and animal health, a limited diagnostic capacity, and weak regulatory enforcement. To address this challenge, Standard Treatment Guidelines (STGs) were developed for the veterinary sector, which represents a major milestone in the country’s AMR containment strategy. STGs are evidence-based protocols that guide veterinary professionals in consistently and appropriately diagnosing and treating animal diseases. They promote the rational use of veterinary medicines, and can mitigate AMR and improve animal health outcomes. By translating the best evidence into best practices, STGs also provide a practical foundation for antimicrobial stewardship (AMS) programs. Until 2023, Zambia lacked nationally adopted STGs for the veterinary sector. The introduction and standardization of these guidelines are expected to promote prudent antimicrobial use and raise the standard of care delivered to animal patients across the country. **Objective**: The aim of this paper is to provide a practical reference for future revisions of STGs and outline the successful methodology used to create STGs in the Zambian animal sector. **Methods**: A situational analysis was conducted to identify priority animal health conditions and existing treatment gaps within the veterinary sector. A multidisciplinary expert committee was then formed, comprising veterinarians, veterinary paraprofessionals, academics, regulatory authorities, and private sector stakeholders, to lead the development of the STGs. The process was guided by the WHO methodology for developing treatment guidelines, including a comprehensive review of the clinical evidence, local disease patterns, antimicrobial resistance data, and existing treatment practices. Draft STGs were developed with clearly defined, species-specific treatment protocols tailored to the Zambian context. For the validation process, the AGREE II instrument was used to assess the quality, clarity, and applicability of the guidelines. Structured stakeholder consultations with practitioners, policy-makers, and technical experts were held to ensure that the guidelines were practical, evidence-based, and aligned with national priorities. The validated drafts were then disseminated and piloted in selected districts. **Conclusions**: The development of the species-specific STGs represents an essential turning point in the country’s efforts to promote responsible veterinary care and contain AMR. STGs have become a prominent key support in the delivery of quality animal care. Further, the guidelines will assist in the optimization of antimicrobial use in animal health in Zambia.

## 1. Introduction

The increase in antimicrobial resistance (AMR) is recognized as a threat to modern medicine and public health [1,2,3,4]. In an attempt to control AMR, the responsible use of antimicrobials is warranted, and a decrease in the inappropriate use of antimicrobials is necessary, both in human and veterinary medicine [5]. Veterinarians play a key role in AMS through disease prevention, clinical care, and client education. In veterinary medicine, the term antimicrobial stewardship (AMS) usually encompasses numerous elements directed towards improved antimicrobial use (AMU) [6]. The principles of AMS in animal health focus on promoting the responsible and judicious use of antimicrobials to preserve their effectiveness, safeguard animal welfare, and protect public health [7]. One of the critical steps toward strengthening antimicrobial stewardship (AMS) in veterinary medicine is the development of National Action Plans (NAPs) on AMR that explicitly incorporate veterinary practices. By integrating veterinary medicine into these national strategies, NAPs serve as a foundational framework for guiding coordinated actions, improving antimicrobial use in animals, and ensuring alignment with broader One Health efforts to combat AMR [4].

Another element that is vital in aiding the progression of AMS programs in animal health includes the development of Standard Treatment Guidelines (STGs) [8]. Treatment guidelines translate the AMS principles into practice. The veterinary profession in Zambia had been operating without STGs since the inception of organized veterinary services in the country in 1907. As a result, practitioners have, over the years, relied on guidelines from foreign veterinary jurisdictions and, in other instances, on manufacturers of veterinary medicinal products (VMPs). This situation has not only led to a multiplicity of therapeutic treatment protocols in the country for addressing similar conditions and diseases, but also a marked variation in the quality of veterinary care in the country’s animal health facilities [9,10].

At both national and sub-national levels, disease control is hampered by inconsistent treatment protocols. The absence of STGs plays a key role in the inconsistent use of antimicrobials. Strengthening STG implementation could help improve prescribing practices and support more effective AMS programs. Animal health STGs can simply be defined as evidence-based protocols that guide veterinary professionals in consistently and appropriately diagnosing and treating animal diseases [11]. STGs are designed to promote rational use of veterinary medicines, reduce AMR, and improve animal health and productivity. Evidence-based STGs represent a systematic approach to translating the best available research evidence into clear statements regarding the treatments of animals. The quality of the STGs is based on comprehensive reviews of the research evidence that can help practitioners make better decisions. STGs need to be clear about what actions they are recommending, as recommendations that are vague or nonspecific are likely to be misinterpreted [12].

In the animal health sector, the guidelines are meant to

- Ensure standardized treatment for common animal diseases across veterinary practices.
- Promote the judicious use of antimicrobials, antiparasitics, and vaccines.
- Improve animal welfare, food safety, and public health (especially in zoonotic disease control).
- Strengthen surveillance and reporting systems as a central component of the One Health approach, facilitating the timely detection of antimicrobial resistance, improving data sharing between veterinary and public health authorities, and supporting coordinated interventions across the human, animal, and environmental health sectors.

To address the diverse needs of different animal species, treatment guidelines were developed as separate documents. These included poultry, bovine, fish, ovine and caprine, porcine, and companion (dogs and cats) animals. In developing these STGs, the World Health Organization (WHO) AWaRe (Access, Watch, and Reserve) classification was used as a tool to guide veterinary professionals and para-professionals in the selection and use of antimicrobials, alongside the OIE (WOAH) List of Critically Important Antimicrobials for Veterinary Use, to better support responsible prescribing and antimicrobial stewardship activities. This was done based on previous findings that found that the WHO AWaRe framework can be applied and AMS programs can be instigated in animal health in Zambia [13]. While the application may be different in veterinary medicine and practice, it is recommended that all attending veterinary professionals and para-professionals in both the private and public sectors be familiar with and maintain an updated WHO AWaRe classification of antibiotics for evaluation and monitoring of use. To adapt the WHO AWaRe classification for veterinary medicine, the framework was modified to align with the unique needs and practices of the animal health sector in Zambia. While the core principles of categorizing antimicrobials based on their importance and risk of resistance development were maintained, adjustments were made to reflect veterinary-specific indications, species applicability, and local availability of drugs. The classification was mapped against the WOAH List of Critically Important Antimicrobials for Veterinary Use to ensure relevance and consistency with global veterinary priorities. Antimicrobials commonly used in livestock were reviewed and re-categorized where appropriate—for example, certain drugs classified as “Watch” in human medicine were placed in the “Access” category for veterinary use due to their lower risk profile in animals or limited relevance to human health. Additionally, the framework emphasized withdrawal periods and residue risks, which are particularly critical in veterinary settings. This adapted AWaRe approach provided a context-specific tool to support rational antimicrobial selection and stewardship in veterinary practice while remaining aligned with broader AMR containment goals.

In Zambia, the animal health sector faces several critical gaps that hinder effective antimicrobial stewardship and contribute to the growing threat of AMR. Foremost is the absence of nationally endorsed veterinary STGs to guide evidence-based prescribing and promote the prudent use of antimicrobials. Therefore, this manuscript outlines the successful development of the STGs for the animal health sector in Zambia and will serve as a practical reference for future revisions.

## 2. Discussion

Antimicrobial stewardship in animal health refers to the coordinated efforts to promote the responsible and judicious use of antimicrobial agents such as antibiotics in both humans and animals, with the aim of improving treatment outcomes, reducing AMR, and preserving the effectiveness of existing drugs [14,15,16]. In Zambia, AMS focuses on optimizing antimicrobial use despite limited resources by strengthening regulations, improving awareness and education, ensuring access to quality-assured medicines, promoting infection prevention practices, enhancing diagnostic capacities, and encouraging collaboration across the human, animal, and environmental health sectors through a One Health approach. This document focuses specifically on the animal health sector, recognizing that AMR in this sector involves distinct challenges compared to the human and environmental sectors. In low-income countries like Zambia, AMS in the human and animal health sectors faces overlapping yet distinct challenges. In the human health sector, the barriers are often rooted in weak clinical governance, limited diagnostic capacities, shortages of trained personnel, and patient or prescriber behaviours that favour unnecessary antibiotic use [17,18,19]. In contrast, the animal health sector is more heavily influenced by economic incentives, such as the use of antimicrobials for growth promotion and routine disease prevention, which are compounded by minimal veterinary oversight and poor enforcement of regulations [10,20]. In order to mitigate these challenges, Zambia is implementing pragmatic, resource-sensitive interventions across both the human and animal health sectors. The government has developed a National Action Plan on AMR aligned with the WHO Global Action Plan and has introduced measures to strengthen the regulation of antimicrobial access, including moves toward prescription-only sales, although enforcement remains a work in progress [4]. Capacity building is being advanced through the training of veterinarians and para-professionals, as well as the integration of AMS principles into veterinary curricula. Public awareness campaigns on drug optimization are being conducted to sensitize farmers to preventive practices such as vaccination, improved hygiene, biosecurity and Infection prevention and control measures. Building on these initiatives, the development of STGs represents the most recent AMS intervention in Zambia, providing evidence-based recommendations to optimize antimicrobial use, standardize prescribing practices, and bridge gaps between policy and clinical or veterinary practice. This paper offers a novel policy perspective by systematically identifying the key antimicrobial stewardship challenges specific to Zambia’s animal health sector, including the limited awareness among stakeholders, the inadequate surveillance infrastructure, and resource constraints affecting sampling and data quality. Beyond merely describing these challenges, the paper proposes actionable strategies to overcome them, such as strengthening laboratory capacities, enhancing stakeholder education, and integrating One Health approaches to improve coordination across sectors. By outlining these targeted solutions, this manuscript provides practical guidance for policymakers and practitioners, enabling them to design more effective stewardship interventions.

The developments and implementation of standardized veterinary STGs in Zambia represent a critical policy advancement in the country’s fight against AMR. By aligning with Zambia’s National Action Plan on AMR and global frameworks such as the WHO Global Action Plan and WOAH guidelines, these STGs operationalize national AMS strategies in the veterinary sector, providing a practical tool for harmonizing antimicrobial use across diverse animal species and production systems. The formal integration of the STGs into veterinary professional accreditation and licensing systems exemplifies a policy mechanism that promotes sustained compliance and stewardship accountability. Furthermore, embedding the guidelines within the broader One Health surveillance framework facilitates multisectoral data sharing and coordinated interventions that are essential for comprehensive AMR containment. Despite these gains, policy challenges persist, including limited local surveillance data and resource constraints, underscoring the need for continued government commitment to funding, multisectoral coordination, and legislative support. Sustained policy engagement will also be essential to regularly update the STGs in response to evolving resistance patterns and emerging scientific evidence. Ultimately, this policy-driven approach not only strengthens veterinary antimicrobial stewardship but also contributes significantly to protecting public health and advancing Zambia’s broader AMR goals within the One Health agenda.

## 3. Methods

### 3.1. Preparatory Phase and Stakeholder Engagement

#### 3.1.1. Formation of the Secretariat

A secretariat comprising personnel from the Ministry of Fisheries and Livestock (MoFL) and the Antimicrobial Resistance Coordinating Committee (AMRCC) at the Zambia National Public Health Institute (ZNPHI) was established based on the members’ expertise and experience. This team was tasked with overseeing the development process of various discipline-specific Technical Working Groups (TWGs). In addition to providing technical and administrative oversight, the secretariat ensured that all the guidelines were compiled within the set timelines and led the processes of printing, launching, and disseminating the guidelines.

#### 3.1.2. Establishment of Technical Working Groups (TWGs)

The development of the STGs was supported by funding from various collaborating partners, each employing different approaches to establish Technical Working Groups (TWGs). In some instances, individual consultants were engaged to lead the process and later assembled their own TWGs. In other cases, consultant groups were directly appointed and designated as the TWG from the beginning.

The TWGs comprised representatives from the Department of Veterinary Services (DVS) in the MoFL, veterinary academia, and research institutions; private veterinary practitioners; pharmaceutical experts; and other livestock specialists (Table 1). A group lead was selected from each of the TWGs to become the primary liaison of the group who responded to comments from the funders and other stakeholders. In other groups, the lead consultant took up this responsibility. While this composition served as the standard structure, the specific individuals involved varied depending on the animal species being addressed, with species-specific experts and relevant veterinary professionals brought in to ensure technical accuracy and contextual relevance for poultry, cattle, small ruminants, and pigs. This flexible yet structured approach ensured that the guidelines were grounded in both scientific evidence and practical field realities. Regional representation was actively considered and achieved by including participants from different laboratories located across the country, universities from various provinces, and TWG members drawn from diverse regions.

The funding agencies provided the Terms of Reference (TORs), which provided the job description, time frame, and fees to the TWGs or consultant. These TWGs bore the primary responsibility for determining the methodology and process for developing the STGs and were accountable to their respective funding agencies.

#### 3.1.3. Defining the Scope and Objectives

A harmonization meeting was held with all the TWGs to ensure that the guidelines developed used a standard format and validation process. The format was then shared among the various stakeholders funding the STG development. The scope of the STGs was agreed upon; the key elements are shown in Table 2.

In addition, the methodology for the development process undertaken by each TWG was to be included in the STGs.

### 3.2. Evidence Gathering and Review

A comprehensive literature review was undertaken to identify and characterize the most prevalent animal diseases in Zambia, as well as the antimicrobial susceptibility patterns of key pathogens. In addition to scientific literature, the development process was informed by the practical insights and field experiences of the guideline drafters.

Peer-reviewed scientific publications were sourced from PubMed, Web of Science, and Scopus, while key reports and technical documents from international organizations were reviewed, including the WOAH List of Antimicrobial Agents of Veterinary Importance and Terrestrial Animal Health Code [21], the WHO Critically Important Antimicrobials for Human Medicine [22] and Guidelines on Integrated Surveillance of Antimicrobial Resistance in Foodborne Bacteria [23], and the FAO Manual on Prudent and Efficient Use of Antimicrobials in Pigs and Poultry [24], alongside Standard Treatment Guidelines from other countries. Locally generated AMR data were obtained from five labs: the Central Veterinary Laboratory Institute (CVRI) (National Reference AMR laboratory), Choma Provincial Veterinary Laboratory, Chipata Provincial Veterinary Laboratory, Mongu Provincial Veterinary Laboratory, and the University of Zambia School of Veterinary Medicine Microbiology Laboratory. These laboratories are the designated laboratories for National AMR surveillance in Zambia.

Furthermore, several TWGs conducted targeted surveys among veterinary professionals to gather information on prescribing practices and the availability of veterinary medicines. This multi-pronged approach ensured that the recommendations within the guidelines were grounded in real-world practice, thereby enhancing their relevance, applicability, and credibility among the end users.

### 3.3. Drafting, Review, and Validation

#### 3.3.1. Drafting of Guidelines

During the drafting of the STGs, disease conditions were selected based on their prevalence and public health significance in Zambia, as reported by national animal health surveillance data, epidemiological studies, and stakeholder consultations. Priority was given to conditions with high morbidity and mortality, substantial economic impacts, or relevance to zoonotic transmission. This approach ensured that the guidelines addressed the most pressing animal health issues with a strong focus on antimicrobial stewardship. In addition to disease-specific STGs, some of the TWGs also developed guidelines organized by physiological systems, such as the gastrointestinal or respiratory systems, offering a more holistic and practical reference for veterinary professionals.

A central emphasis throughout the development process was the promotion of responsible antimicrobial use. Furthermore, to enhance the diagnostic accuracy and practical utility of the guidelines, some STGs included detailed guidance on conducting postmortem examinations, particularly in cases where necropsy is commonly employed for disease diagnosis. This was especially relevant in poultry medicine, where postmortem examination findings often play a critical role in identifying and managing disease outbreaks and can be easily conducted.

A common strategy adopted by the TWGs involved distributing responsibilities among their members to accelerate the development of the guidelines. Once the overall approach had been agreed upon and the list of target diseases finalized, tasks were allocated based on each member’s area of expertise and availability. This collaborative division of labour not only enhanced efficiency but also ensured that the development process leveraged a broad range of technical insights within a compressed time frame.

#### 3.3.2. Review and Validation

The draft STGs underwent a rigorous peer-review process involving a broader network of veterinary professionals, academics, and, in some instances, international experts. This step aimed to gather constructive feedback to enhance the content and ensure technical soundness. Following this, a validation workshop was convened to facilitate in-depth discussions, clarify ambiguities, and refine the guidelines to ensure their clarity, accuracy, practical applicability, and local relevance.

As part of the quality assurance process, the draft guidelines were evaluated using the Appraisal of Guidelines for Research & Evaluation II (AGREE II) instrument [25]. This tool provided a structured framework for assessing the methodological rigour, transparency, and overall quality of the guidelines, ensuring that the final document met recognized international standards. Following the validation process, the TWGs produced the final drafts of the guidelines for professional formatting and printing.

### 3.4. Launch and Dissemination

After the validation process, the STGs were officially launched and made available to practitioners in the country. Following the national dissemination meeting, a piloting program was initiated to introduce the newly developed STGs to veterinary professionals across selected districts in Zambia. The first phase involved targeted engagement with veterinarians, during which the STGs were presented and their structure, purpose, and importance in promoting rational antimicrobial use and supporting antimicrobial stewardship were explained in detail. Veterinarians were trained on how to interpret and apply the guidelines to clinical decision-making in various livestock production systems. After this orientation, each veterinarian was tasked with cascading the training to their respective teams of veterinarians, veterinary assistants, and para-professionals within their districts. This approach aimed to foster a peer-led understanding of the STGs, promote ownership at the district level, and ensure that the guidelines reached frontline animal health service providers. Feedback mechanisms were established to capture initial user experiences, identify implementation challenges, and gather suggestions for refining the guidelines before the nationwide roll-out. To promote sustained adherence to STGs, compliance will be incentivized through integration into professional accreditation and license renewal requirements.

### 3.5. Review Period

The STGs were scheduled for review every two years to accommodate developments within the veterinary sector, evolving antimicrobial resistance patterns, and changes in the availability of veterinary medicines. This periodic review also aims to ensure continued alignment with emerging global standards and best practices.

### 3.6. AMS Challenges in the Country

The Zambian animal health sector faces multiple challenges that hinder effective AMS and contribute to antimicrobial resistance. Low awareness and understanding of AMS among livestock farmers, agrovet workers, and some veterinary professionals result in misuse and overuse of antimicrobials [9,13,26]. Weak regulatory enforcement allows for frequent over-the-counter sales of antibiotics without prescriptions or proper guidance [10]. Limited access to veterinary services, especially in rural areas, often drives farmers to self-medicate or rely on unqualified personnel [20]. The scarcity of routine diagnostic testing and inadequate laboratory infrastructure has led to empirical antimicrobial use without pathogen confirmation or drug sensitivity data [20]. Surveillance systems for monitoring antimicrobial use and resistance remain underdeveloped, limiting evidence-based policy and stewardship decisions. Economic pressures in intensive farming systems exert influence on both farmers and veterinary professionals, with implications for antimicrobial stewardship. Farmers, under constant pressure to maximize productivity and profitability, may prefer rapid, low-cost interventions such as non-therapeutic antibiotic use over long-term preventive measures. Veterinary professionals, meanwhile, are influenced by client expectations for immediate outcomes, a reliance on medicine sales for income, and limited access to affordable diagnostic services. These combined pressures can inadvertently incentivize inappropriate antimicrobial use, highlighting the need for policy interventions such as strengthening veterinary service financing models, expanding access to diagnostics, and promoting preventive animal health strategies to break this cycle and align economic incentives with responsible antimicrobial use.

Despite the implementation of other AMS measures, the development of STGs remain a critical intervention in Zambia’s veterinary antimicrobial stewardship programme because they provide a structured, evidence-based framework for clinical decision-making across diverse settings. Unlike other stewardship measures such as awareness campaigns, biosecurity improvements, or vaccination programmes, which are essential but often indirect in their impact on prescribing behaviour, STGs directly influence antimicrobial use at the point of care. They reduce variability in clinical practice, promote the prudent selection, dosage, and duration of antimicrobial use, and ensure alignment with both international recommendations and local epidemiological data. This direct, standardized approach bridges the gap between policy and practice, enabling consistent application of best practices nationwide. In Zambia, where veterinary services are often decentralized and antimicrobial access is variable, STGs offer a unifying, authoritative reference that can be applied across regions, making them a critical tool for achieving measurable and sustainable reductions in inappropriate antimicrobial use.

The development of the veterinary STGs faced challenges including limited local AMR surveillance data, with most existing data being unpublished and having limited sample sizes, which constrains the formulation of species-specific recommendations. To address these issues, efforts are underway to strengthen the national AMR surveillance system by increasing the laboratory network to capture more data. Further efforts are being made to enhance active surveillance for poultry, cattle, and pigs by developing active surveillance protocols based on WOAH and FAO guidelines. The diagnostic capacity is being enhanced via infrastructure upgrades, improvements in reagent availability, and personnel training in advanced AMR detection methods. Capacity building targeting veterinary para-professionals through workshops and mentorship focused on STGs, disease recognition, sample collection, biosecurity, and antimicrobial stewardship is also underway.

## 4. Conclusions

In order to operationalize the STGs effectively, nationwide training programs will be essential to build the capacity of veterinarians, veterinary para-professionals, and agrovet personnel. These training programs will focus on guideline implementation, appropriate drug selection and dosage, and adherence to withdrawal periods. Integrating the STGs with the national veterinary drug supply system will be a critical next step, ensuring that recommended antimicrobials are not only available but also prioritized according to their classification under the WHO AWaRe framework and the OIE List of Critically Important Antimicrobials for Veterinary Use. Periodic compliance audits are recommended to monitor the use of STGs and ensure alignment with best practices. The development of the species-specific STGs represents an essential turning point in the country’s efforts to promote responsible veterinary care and contain antimicrobial resistance. The guidelines created the foundation for a more robust, effective, and accountable animal health system that benefits the public, veterinarians, and livestock owners by incorporating best practices into routine operations. The guidelines will also reduce unjustified variation in the care delivered.

Lastly, the process of STG development highlighted the areas in which the evidence is lacking in quantity or quality. It is also important to note that some treatments may have been left out due to lack of evidence; however, the absence of evidence is not evidence of absence. We believe the development of these guidelines will contribute to the effective implementation of AMS programs in the animal health sector in Zambia.

## Figures and Tables

**Table 1:** Composition of the STG development group.

Category	Number of Members	Selection Criteria
AMRCC Technical Working Group (TWG) Members (2 from each)	10	Nominated from each of the five AMRCC TWGs based on experience in AMR/AMU policy and implementation. The TWGs included Surveillance, Infection Prevention and Control (IPC), Laboratory, Antimicrobial Stewardship (AMS), and Awareness/Education groups
Academia	2	Experts in veterinary pharmacology and public health, nominated by leading universities
Private Veterinary Sector	2	Practicing veterinarians with experience in large-scale production and smallholder systems
Species-Specific Experts	4	Selected for their expertise in poultry, swine, cattle, and small ruminants
Veterinary Pharmaceutical Industry	2	Representatives from registered veterinary pharmaceutical distributors
Zambia Medicines Regulatory Authority (ZAMRA)	2	Officials responsible for veterinary medicine regulation and inspection
Regional Veterinary Laboratories	5	Heads or senior scientists from each of the five regional veterinary laboratories
Department of Veterinary Services (DVS)	4	Senior officers from policy, field services, epidemiology, and AMR coordination units

**Table 2:** Scope with key elements and components included in the STGs.

No	Key Element	Components
1	Disease Overview	Aetiology Transmission Species affected
2	Clinical Signs and Diagnosis	Clinical presentation and diagnostic methods
3	Treatment Options	First-line and second-line treatments and AWARE classification of antimicrobials
		Alternatives in case of resistance and contraindications
		Other medications
		Supportive therapy
4	Withdrawal period	Cautions, especially for animals produced for consumption
5	Preventive measures and client education	Vaccination schedules
		Biosecurity
		Nutrition and hygiene
6	Referral and Reporting guidelines	When to refer cases
		Reporting of notifiable diseases to veterinary authorities

## Data Availability

The data supporting the reported process of developing the STGs can be made available on request from the corresponding author.

## Abbreviations

AMR	Antimicrobial Resistance
AMU	Antimicrobial Use
AMS	Antimicrobial Stewardship
AMRCC	Antimicrobial Resistance Coordinating Committee
AWaRe	Access Watch Reserve
CVRI	Central Veterinary Research Institute
DVS	Department of Veterinary Services
FAO	Food Agriculture Organization
TORs	Terms of Reference
TWG	Technical Working Group
WHO	World Health Organization
WOAH	World Organisation for Animal Health
ZNPHI	Zambia National Public Health Institute
